# Avoiding false discovery in biomarker research

**DOI:** 10.1186/s12858-016-0073-x

**Published:** 2016-07-30

**Authors:** Pranali Patel, Uros Kuzmanov, Seema Mital

**Affiliations:** 1 Program in Genetics and Genome Biology, The Hospital for Sick Children, Toronto, ON Canada; 2 Donnelly Centre for Cellular and Biomolecular Research, University of Toronto, Toronto, ON Canada; 3 Department of Pediatrics, The Hospital for Sick Children, University of Toronto, Toronto, ON Canada; 4 Ted Rogers Centre for Heart Research, Toronto, ON Canada

**Keywords:** ELISA assay, Validation, Biomarker discovery, SIRPA, Mass spectrometry

## Abstract

**Background:**

Human tyrosine-protein phosphatase non-receptor type substrate 1α (SIRPA) is a surface marker identified in cardiomyocytes differentiated from human embryonic stem cells. Our objective was to determine if circulating SIRPA levels can serve as a biomarker of cardiac injury in children undergoing open heart surgery.

**Results:**

Paired pre- and post-operative serum samples from 48 pediatric patients undergoing open heart surgery and from 6 pediatric patients undergoing non-cardiac surgery (controls) were tested for SIRPA protein levels using commercially available SIRPA ELISA kits from two manufacturers. Post-operative SIRPA concentrations were significantly higher in patients after cardiac surgery compared to non-cardiac surgery when tested using SIRPA ELISA kits from both manufacturers. To verify the identity of the protein detected, recombinant human SIRPA protein (rhSIRPA) was tested on both ELISA kits. The calibrator from both ELISA kits was analyzed by Western blot as well as by Mass Spectrometry (MS). Western blot analysis of calibrators from both kits did not identity SIRPA. MS analysis of calibrators from both ELISA kits identified several inflammatory markers and albumin but no SIRPA was detected.

**Conclusions:**

We conclude that commercially available ELISA kits for SIRPA give false-positive results. Verifying protein identity using robust protein characterization is critical to avoid false biomarker discovery when using commercial ELISA kits.

**Electronic supplementary material:**

The online version of this article (doi:10.1186/s12858-016-0073-x) contains supplementary material, which is available to authorized users.

## Background

The need for a robust cardiac biomarker for diagnosis, risk stratification and tailored management of patients with heart disease has resulted in considerable investment by both academic and industry researchers in the search for a novel predictive biomarker that is sensitive, specific and accurate. Although numerous cardiac biomarkers have been identified in recent years and immunoassays have been developed, only a handful have been successfully validated for clinical application due to failure along the various stages of the biomarker discovery pipeline and/or their limited clinical impact on disease prediction and risk stratification [1, 2].

Cardiac troponin is widely used as a sensitive biomarker of cardiac injury. However, it is not specific to mechanism of injury, nor predictive of outcomes and is often disproportionately elevated in renal insufficiency [3]. SIRPA (Signal-regulatory protein alpha or Human tyrosine-protein phosphatase non-receptor type substrate 1 or SHPS or CD172a) is a ~90 kDa transmembrane protein abundantly expressed in neurons, macrophages and dendritic cells [4]. Along with other SIRP (Signal Regulatory protein) family members, SIRPβ and SIRPγ, SIRPA is located on human chromosome 20p13 [5, 6]. SIRPA is involved in various biological processes, including negative regulation of immune cells and suppression of anchorage-independent proliferative signal [7, 8]. SIRPA was first identified as a cardiomyocyte-specific surface marker using high-throughput flow cytometry screening of cardiovascular cell lineages derived from human embryonic stem cells (hESCs) against a panel of 370 known cluster of differentiation antibodies [9]. A role for SIRPA in cardiac diseases is not fully established except for one study showing a protective role of SIRPA in cardiac hypertrophy through negative regulation of the Toll-like receptor 4/nuclear factor-kB pathway in vitro [10]. Our objective was to determine if circulating serum SIRPA concentrations are increased in children undergoing open heart surgery and whether this represents a sensitive and specific marker of cardiomyocyte injury. Our study revealed an increase in post-operative circulating SIRPA concentrations measured using commercial ELISA kits. Unfortunately, subsequent protein validation studies failed to confirm the identity of the detected protein as SIRPA highlighting a major pitfall in biomarker discovery research utilizing commercial ELISA kits.

## Methods

### Study cohort

Children with heart disease undergoing cardiac and non-cardiac surgery were recruited into the Heart Centre Biobank Registry [11]. The study was approved by the Research Ethics Board of the Hospital for Sick Children. Written informed consent to participate and publish results was obtained from all parents/legal guardians/participants. Serum samples were collected prior to cardiac surgery and on post-operative days 1 and 2. Serum samples were stored at 4 °C for 24–48 h and then frozen at -80 °C until analysis. Demographic data, diagnosis, surgery type, and cardiopulmonary bypass duration were collected from medical records. Patients were categorized by presence or absence of a left ventricular (LV) ventriculotomy (incision in LV), or myectomy (resection of myocardium from the LV). Patients undergoing non-cardiac surgery i.e. liver or renal transplant were included as controls.

### ELISA for SIRPA

All serum samples were analyzed for SIRPA concentration using commercially available Sandwich-SIRPA ELISA kits purchased from Cusabio Biotech Co, Ltd, Wuhan, China (Catalog # CSB-EL021334HU) and Elabscience- Biotech Co.Ltd, Wuhan, China (Catalog # E-EL-H1573). ELISA assays on pre- and post-operative serum were performed according to manufacturer’s instructions. After assay, Optical Density (OD) was measured using Gemini EM microplate reader (Molecular Devices, USA) set at 450 nm and change in SIRPA concentrations after surgery were compared between the different surgical groups.

### Western blot analysis of SIRPA ELISA kit calibrators

To verify the identity of the protein detected, Western blot was performed on the calibrators from Cusabio and Elabscience ELISA kits and compared to recombinant human SIRPA (rhSIRPA) which was purchased from Elabscience (Catalog # PN201773) to use as positive control. Briefly, equal amount of protein from each sample was separated by gel electrophoresis using Novex 10 % Tris-Glycine Gel (Life technologies, catalog no# EC6078Box) following manufacturer’s instructions. Protein was transferred to PVDF membrane and blocked with 5 % milk in TBST for 1 h. at room temperature followed by overnight incubation with anti-human SIRPA antibody (Biolegend, catalog # 323805) followed by 1 h incubation with horseradish peroxidase-conjugated secondary antibody at room temperature.

### Mass spectrometry analysis of ELISA kit calibrators

SIRPA ELISA kit calibrators from Cusabio and Elabscience were also analyzed on an Orbitrap analyzer (Q-Exactive, ThermoFisher, San Jose, CA) outfitted with a nanospray source and EASY-nLC nano-LC system (ThermoFisher, San Jose, CA) in order to determine if SIRPA was the target antigen for both calibrators. This was performed at the SickKids SPARC Biocentre (Toronto, ON). The samples were trypsin digested and lyophilized peptide mixtures were dissolved in 0.1 % formic acid and loaded onto a 75 μm x 50 cm PepMax RSLC EASY-Spray column filled with 2 μM C18 beads (ThermoFisher San, Jose CA) at a pressure of 800 Bar. Peptides were eluted over 60 mins at a rate of 250 nl/min using a 0 to 35 % acetonitrile gradient in 0.1 % formic acid. Peptides were introduced by nano-electrospray into the Q-Exactive mass spectrometer (Thermo-Fisher). The instrument method consisted of one MS full scan (400–1500 m/z) in the Orbitrap mass analyzer with an automatic gain control (AGC) target of 1e6, maximum ion injection time of 120 ms and a resolution of 70,000 followed by 10 data-dependent MS/MS scans with a resolution of 17,500, an AGC target of 1e6, maximum ion time of 120 ms, and one microscan. The intensity threshold to trigger a MS/MS scan was set to 1.7e4. Fragmentation occurred in the Higher –energy C-trap dissociation with normalized collision energy set to 27. The dynamic exclusion was applied using a setting of 5 s.

### Statistical analysis

Serum SIRPA concentrations were expressed as mean ± standard deviation. Samples were grouped by the type of cardiac surgery i.e. no-ventriculotomy (*n* = 27), myectomy (*n* = 14), and ventriculotomy (*n* = 7). Samples from non-cardiac surgery i.e. liver and renal transplant (*n* = 6) were used as a controls. Statistical analysis was performed by Student *t*-test to compare SIRPA concentrations in cases and controls and to compare change in SIRPA concentrations in paired samples from pre- to post-operative time-points. Differences were considered statistically significant if *p* < 0.05. GraphPad PRISM 6.05 (GraphPad software, USA) was used for statistical analysis.

## Results

### Elevated post-operative SIRPA concentrations using ELISA

**Fig. 1:**
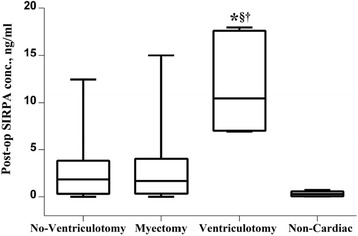
Post-operative serum SIRPA concentration from 54 patients using Cusabio SIRPA ELISA kit. Patients were grouped into no ventriculotomy (*n* = 28), myectomy (*n* = 14), ventriculotomy (*n* = 7) and non-cardiac surgery i.e. liver and renal transplants as negative controls (*n* = 6). Post-operative serum SIRPA concentrations were significantly higher in patients receiving ventriculotomy compared to other groups (*p < 0.0001 vs no ventriculotomy, §*p* = 0.0004 vs myectomy and †*p* = 0.0001 vs non-cardiac)

Paired sera (*n* = 108), pre-operative and post-operative, from 54 patients were used to measure serum SIRPA concentration using Cusabio SIRPA ELISA kit. Post-operative serum SIRPA concentrations were significantly higher in patients receiving ventriculotomy compared to patients not receiving ventriculotomy (*p* < 0.0001), or myectomy (*p* = 0.0004) and compared to those undergoing non-cardiac surgery (*p* = 0.0001) (Fig. 1). To replicate findings using another assay kit, we analyzed 4 serum samples (paired sera) from 2 patients using a SIRPA ELISA assay kit from ElabScience, China. This assay also detected a post-operative increase (0.184 ± 0.005 ng/ml and 5.104 ± 0.253 ng/ml) in serum SIRPA concentrations compared to pre-operative concentrations (0 ng/ml for both samples tested). To verify the identity of the detected protein, we performed additional experiments.

### SIRPA ELISA kits failed to recognize pure rhSIRPA protein

**Fig. 2:**
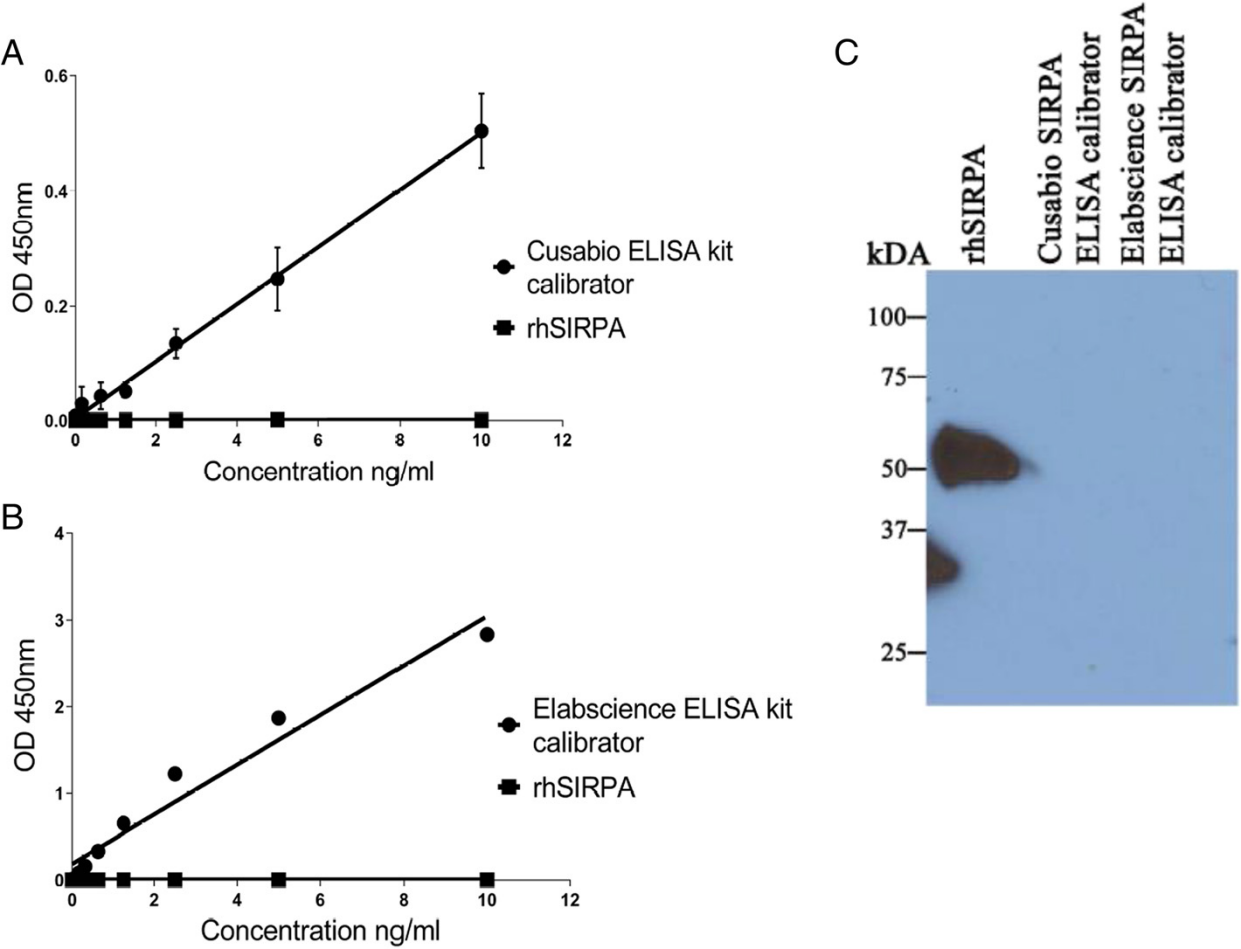
Cusabio and Elabscience SIRPA ELISA kits failed to recognize recombinant human SIRPA protein. **a** Cusabio SIRPA ELISA kit (CSB-EL021334HU) recognized its own calibrator diluted in buffer from Cusabio ELISA kit generating a linear curve (*diamonds*) but did not recognize recombinant human SIRPA protein (*squares*); **b** Elabscience SIRPA ELISA kit (E-EL-H1573) recognized its own calibrator diluted in buffer from Elabscience ELISA kit generating a linear curve (*diamonds*) but did not recognize recombinant human SIRPA protein (*squares*). X- axis represents known standard concentration (ng/ml) and Y-axis represents optical density (OD) measured at 450 nm. **c** Western blot of rhSIRPA using SIRPA antibody (Biolegend, catalog # 323805) detected a ~55 kda band in rhSIRPA but not in calibrators from Cusabio and Elabscience SIRPA ELISA kits

We purchased full length, rhSIRPA protein from Elabscience in order to verify that the protein being detected using ELISA was SIRPA. rhSIRPA consists of 504 amino acids and predicts a molecular mass of 55 kDa in its native form and 60-65 kDA under reducing conditions due to glycosylation. As a first step, we verified using MS that the rhSIRPA from Elabscience was indeed SIRPA (see Additional file 1: Table S1). After this verification, we examined the ability of the Cusabio and Elabscience ELISA kits to detect known quantities of full length rhSIRPA using serial known dilutions of rhSIRPA (0.156-10 ng/ml). As shown in Fig. 2a and b, the OD 450 nm readings from both assays were close to zero indicating that the assays do not recognize full length rhSIRPA protein (Table 1) even though both ELISA kits were able to recognize their respective kit calibrators, producing linear calibration curves (Fig. 2a and b).

**Table 1:** Mass spectrometry identification and ELISA immunoreactivity of SIRPA

Sample	SIRPA ELISA immunoreactivity (Cusabio)	SIRPA ELISA immunoreactivity (Elabscience)	MS identification of SIRPA
rhSIRPA protein	No	No	Yes
Calibrator from Cusabio ELISA kit	Yes	No	No
Calibrator from Elabscience ELISA kit	Not tested	Yes	No

### Western blot analysis of SIRPA ELISA kit calibrators

We then performed Western blot analysis on the leftover calibrators we could use from both ELISA kits using anti-human SIRPA antibody from Biolegend (catalog # 323805) that has been previously used successfully to detect SIRPA-positive cardiomyocytes [9]. As shown in Fig. 2c, we detected a band of approximately 55 kDa with rhSIRPA protein but not with either of the calibrators suggesting that both calibrators do not react with SIRPA antibody.

### MS analysis of SIRPA ELISA kit calibrators

We then performed MS analysis of Cusabio and Elabscience ELISA kit calibrators with rhSIRPA as a positive control. rhSIRPA was readily detected using MS (Additional file 1: Table S1). However, SIRPA was not detected in either ELISA kit calibrator. Instead, the calibrators were found to contain mostly albumin and a large number of other proteins, including inflammatory proteins (Additional file 1: Tables S2 and S3).

## Discussion

In this report, we highlight the perils in biomarker discovery when using protein assays that are poorly validated. We report a case of poorly characterized ELISA kits purchased from two manufacturers that identify antigens unrelated to the target analyte. Despite initial findings of a post-operative increase in circulating SIRPA protein in patients undergoing ventriculotomy on ELISA testing from two manufacturers, extensive validation experiments failed to replicate this finding. ELISA kits from both manufacturers failed to recognize full length rhSIRPA protein (MS verified, Table 1). MS analysis of kit calibrators revealed that several inflammatory proteins in addition to albumin were identified instead of SIRPA (see Additional file 1: Tables S2 and S3). Even after repeated request, we were unable to purchase/obtain calibrators from the ELISA kit manufacturers for further experiments.

Of note, Elabscience ELISA kit was unable to recognize rhSIRPA protein manufactured by its own company (Fig. 2b) which the company attributed to “spatial conformational change in rhSIRPA that may interfere with its recognition by the ELISA kit”. Unfortunately, this highlights poor analytical validation of the product at the source. To our knowledge, this is at least the third documented evidence of poorly validated commercial ELISA kits that do not recognize the intended antigen. A similar situation was encountered by Prassas et al. [12], where they showed that a commercial ELISA kit purchased from USCN life science (Wuhan, China) that was marketed for the analyte, CUZD1, recognized a different non-homologous antigen, CA125. The authors indicated that many commercial manufacturers acquire their reagent from external suppliers without rigorous quality assurance. In another study, an ELISA assay from USCN Life Sciences, designed to analyze soluble hemojuvelin in humans was unable to quantify human hemojuvelin but detected a different unknown antigen [13].

To explore the validity of SIRPA as a biomarker of cardiac injury will require development of a sensitive mass spectrometry based targeted method for serum samples as no other validated assay is currently available. Also, we did not perform experiments to determine limits of detection of SIRPA in human serum since this was beyond the scope of our current study.

## Conclusion

The growing interest in biomarker discovery research for new diagnostic and therapeutic targets has seen the market flooded with antibodies and ELISA kits from vendors that are marketed “for Research purposes only”. These commercially available assays offer an attractive option to rapidly screen for candidate proteins in large sample sets due to ease of availability, ease of operation, ability to test multiple samples and/or multiplex assays. However, our report and other reports suggest that caution should be exercised when using commercial research grade ELISA kits because they may be incompletely characterized and validated, and results from these assays should be interpreted with extreme caution until systematic and independent validation is performed to verify the findings [14, 15]. This is critical in order to avoid the risk of false biomarker discovery and chasing spurious targets.

## Abbreviations

hESCs, human embryonic stem cells; LV, left ventricle; MS, mass spectrometry; OD, optical density; rhSIRPA, recombinant human SIRPA; SIRPA, human tyrosine-protein phosphatase non-receptor type substrate 1α or Signal-regulatory protein alpha

## Additional file

Additional file 1: Table S1. Mass spectrometry analysis of rhSIRPA protein. **Table S2.** Mass spectrometry analysis of calibrator from SIRPA ELISA kit (Cusabio). **Table S3.** Mass spectrometry analysis of calibrator from Elabscience SIRPA ELISA kit. (DOCX 37 kb)
