# Supplementary material for: Avoiding false discovery in biomarker research
Source: BMC Biochem. 2016 Jul 30;17:17. doi: 10.1186/s12858-016-0073-x (PMC4967310; doi:10.1186/s12858-016-0073-x)
Supplement: Additional file 1: Table S1. — Mass spectrometry analysis of rhSIRPA protein. Table S2. Mass spectrometry analysis of calibrator from SIRPA ELISA kit (Cusabio). Table S3. Mass spectrometry analysis of calibrator from Elabscience SIRPA ELISA kit. (DOCX 37 kb) [file 12858_2016_73_MOESM1_ESM.docx]

**Additional files**

**Additional file 1: Table S1**. Mass spectrometry analysis of rhSIRPA protein

| **Accession** | **-10lgP** | **Coverage (%)** | **#Peptides** | **#Unique** | **Avg. Mass** | **Description** |
| --- | --- | --- | --- | --- | --- | --- |
| P78324\|SHPS1_HUMAN | 437.54 | 65 | 83 | 29 | 54967 | Tyrosine-protein phosphatase non-receptor type substrate 1 OS=Homo sapiens GN=SIRPA PE=1 SV=2 |
| O00241\|SIRB1_HUMAN | 241.96 | 20 | 14 | 1 | 43211 | Signal-regulatory protein beta-1 OS=Homo sapiens GN=SIRPB1 PE=1 SV=5 |
| P08238\|HS90B_HUMAN | 230.71 | 21 | 12 | 6 | 83264 | Heat shock protein HSP 90-beta OS=Homo sapiens GN=HSP90AB1 PE=1 SV=4 |
| P08107\|HSP71_HUMAN | 224 | 21 | 12 | 10 | 70052 | Heat shock 70 kDa protein 1A/1B OS=Homo sapiens GN=HSPA1A PE=1 SV=5 |
| P68363\|TBA1B_HUMAN | 221.76 | 31 | 9 | 9 | 50152 | Tubulin alpha-1B chain OS=Homo sapiens GN=TUBA1B PE=1 SV=1 |
| Q9P1W8\|SIRPG_HUMAN | 213.25 | 21 | 18 | 1 | 42498 | Signal-regulatory protein gamma OS=Homo sapiens GN=SIRPG PE=1 SV=3 |
| P04264\|K2C1_HUMAN | 205.58 | 22 | 13 | 13 | 66039 | Keratin, type II cytoskeletal 1 OS=Homo sapiens GN=KRT1 PE=1 SV=6 |
| P07900\|HS90A_HUMAN | 196.72 | 18 | 10 | 4 | 84660 | Heat shock protein HSP 90-alpha OS=Homo sapiens GN=HSP90AA1 PE=1 SV=5 |
| P13645\|K1C10_HUMAN | 192.59 | 27 | 12 | 9 | 58827 | Keratin, type I cytoskeletal 10 OS=Homo sapiens GN=KRT10 PE=1 SV=6 |
| P63261\|ACTG_HUMAN | 189.36 | 32 | 8 | 3 | 41793 | Actin, cytoplasmic 2 OS=Homo sapiens GN=ACTG1 PE=1 SV=1 |
| P60709\|ACTB_HUMAN | 189.36 | 32 | 8 | 3 | 41737 | Actin, cytoplasmic 1 OS=Homo sapiens GN=ACTB PE=1 SV=1 |
| P68104\|EF1A1_HUMAN | 171.93 | 15 | 5 | 5 | 50141 | Elongation factor 1-alpha 1 OS=Homo sapiens GN=EEF1A1 PE=1 SV=1 |
| Q5VTE0\|EF1A3_HUMAN | 171.93 | 15 | 5 | 5 | 50185 | Putative elongation factor 1-alpha-like 3 OS=Homo sapiens GN=EEF1A1P5 PE=5 SV=1 |
| Q9GZN8\|CT027_HUMAN | 166.84 | 45 | 5 | 5 | 19291 | UPF0687 protein C20orf27 OS=Homo sapiens GN=C20orf27 PE=1 SV=3 |
| P68133\|ACTS_HUMAN | 159.48 | 20 | 6 | 1 | 42051 | Actin, alpha skeletal muscle OS=Homo sapiens GN=ACTA1 PE=1 SV=1 |
| P68032\|ACTC_HUMAN | 159.48 | 20 | 6 | 1 | 42019 | Actin, alpha cardiac muscle 1 OS=Homo sapiens GN=ACTC1 PE=1 SV=1 |
| P07437\|TBB5_HUMAN | 141.26 | 14 | 5 | 5 | 49671 | Tubulin beta chain OS=Homo sapiens GN=TUBB PE=1 SV=2 |
| P54652\|HSP72_HUMAN | 132.66 | 11 | 5 | 3 | 70021 | Heat shock-related 70 kDa protein 2 OS=Homo sapiens GN=HSPA2 PE=1 SV=1 |
| P11142\|HSP7C_HUMAN | 132.66 | 11 | 5 | 3 | 70898 | Heat shock cognate 71 kDa protein OS=Homo sapiens GN=HSPA8 PE=1 SV=1 |
| O76061\|STC2_HUMAN | 126.99 | 14 | 4 | 4 | 33249 | Stanniocalcin-2 OS=Homo sapiens GN=STC2 PE=1 SV=1 |
| Q15393\|SF3B3_HUMAN | 123.19 | 5 | 5 | 5 | 135577 | Splicing factor 3B subunit 3 OS=Homo sapiens GN=SF3B3 PE=1 SV=4 |
| P35527\|K1C9_HUMAN | 118.81 | 9 | 5 | 5 | 62064 | Keratin, type I cytoskeletal 9 OS=Homo sapiens GN=KRT9 PE=1 SV=3 |
| Q9NTM9\|CUTC_HUMAN | 100.93 | 12 | 2 | 2 | 29341 | Copper homeostasis protein cutC homolog OS=Homo sapiens GN=CUTC PE=1 SV=1 |
| P30566\|PUR8_HUMAN | 100.23 | 6 | 3 | 3 | 54889 | Adenylosuccinate lyase OS=Homo sapiens GN=ADSL PE=1 SV=2 |
| P28799\|GRN_HUMAN | 95.3 | 7 | 3 | 3 | 63545 | Granulins OS=Homo sapiens GN=GRN PE=1 SV=2 |
| Q15459\|SF3A1_HUMAN | 95.1 | 3 | 2 | 2 | 88886 | Splicing factor 3A subunit 1 OS=Homo sapiens GN=SF3A1 PE=1 SV=1 |
| Q12874\|SF3A3_HUMAN | 86.59 | 4 | 2 | 2 | 58849 | Splicing factor 3A subunit 3 OS=Homo sapiens GN=SF3A3 PE=1 SV=1 |
| Q86TI2\|DPP9_HUMAN | 84.32 | 3 | 3 | 3 | 98263 | Dipeptidyl peptidase 9 OS=Homo sapiens GN=DPP9 PE=1 SV=3 |
| O75533\|SF3B1_HUMAN | 81.32 | 2 | 2 | 2 | 145830 | Splicing factor 3B subunit 1 OS=Homo sapiens GN=SF3B1 PE=1 SV=3 |
| P04406\|G3P_HUMAN | 79.4 | 9 | 2 | 2 | 36053 | Glyceraldehyde-3-phosphate dehydrogenase OS=Homo sapiens GN=GAPDH PE=1 SV=3 |
| P06733\|ENOA_HUMAN | 77.1 | 7 | 3 | 3 | 47169 | Alpha-enolase OS=Homo sapiens GN=ENO1 PE=1 SV=2 |
| P13639\|EF2_HUMAN | 76.16 | 2 | 2 | 2 | 95338 | Elongation factor 2 OS=Homo sapiens GN=EEF2 PE=1 SV=4 |
| P00558\|PGK1_HUMAN | 73.78 | 4 | 1 | 1 | 44615 | Phosphoglycerate kinase 1 OS=Homo sapiens GN=PGK1 PE=1 SV=3 |
| P07205\|PGK2_HUMAN | 73.78 | 4 | 1 | 1 | 44796 | Phosphoglycerate kinase 2 OS=Homo sapiens GN=PGK2 PE=1 SV=3 |
| Q9Y4L1\|HYOU1_HUMAN | 70.11 | 3 | 2 | 2 | 111335 | Hypoxia up-regulated protein 1 OS=Homo sapiens GN=HYOU1 PE=1 SV=1 |
| Q15427\|SF3B4_HUMAN | 68.44 | 3 | 1 | 1 | 44386 | Splicing factor 3B subunit 4 OS=Homo sapiens GN=SF3B4 PE=1 SV=1 |
| O60361\|NDK8_HUMAN | 65.06 | 7 | 1 | 1 | 15529 | Putative nucleoside diphosphate kinase OS=Homo sapiens GN=NME2P1 PE=5 SV=1 |
| P15531\|NDKA_HUMAN | 65.06 | 6 | 1 | 1 | 17149 | Nucleoside diphosphate kinase A OS=Homo sapiens GN=NME1 PE=1 SV=1 |
| P22392\|NDKB_HUMAN | 65.06 | 6 | 1 | 1 | 17298 | Nucleoside diphosphate kinase B OS=Homo sapiens GN=NME2 PE=1 SV=1 |
| Q01433\|AMPD2_HUMAN | 63.61 | 2 | 2 | 2 | 100688 | AMP deaminase 2 OS=Homo sapiens GN=AMPD2 PE=1 SV=2 |
| P52655\|TF2AA_HUMAN | 63.27 | 3 | 1 | 1 | 41514 | Transcription initiation factor IIA subunit 1 OS=Homo sapiens GN=GTF2A1 PE=1 SV=1 |
| P14618\|KPYM_HUMAN | 59.5 | 2 | 1 | 1 | 57937 | Pyruvate kinase PKM OS=Homo sapiens GN=PKM PE=1 SV=4 |
| P30613\|KPYR_HUMAN | 59.5 | 2 | 1 | 1 | 61830 | Pyruvate kinase PKLR OS=Homo sapiens GN=PKLR PE=1 SV=2 |
| P22314\|UBA1_HUMAN | 56.74 | 1 | 1 | 1 | 117849 | Ubiquitin-like modifier-activating enzyme 1 OS=Homo sapiens GN=UBA1 PE=1 SV=3 |
| P07477\|TRY1_HUMAN | 56.61 | 4 | 1 | 1 | 26558 | Trypsin-1 OS=Homo sapiens GN=PRSS1 PE=1 SV=1 |
| Q8NHM4\|TRY6_HUMAN | 56.61 | 4 | 1 | 1 | 26539 | Putative trypsin-6 OS=Homo sapiens GN=TRY6 PE=5 SV=1 |
| Q15102\|PA1B3_HUMAN | 56.39 | 4 | 1 | 1 | 25734 | Platelet-activating factor acetylhydrolase IB subunit gamma OS=Homo sapiens GN=PAFAH1B3 PE=1 SV=1 |
| Q15428\|SF3A2_HUMAN | 53.77 | 2 | 1 | 1 | 49256 | Splicing factor 3A subunit 2 OS=Homo sapiens GN=SF3A2 PE=1 SV=2 |
| P12277\|KCRB_HUMAN | 53.4 | 3 | 1 | 1 | 42644 | Creatine kinase B-type OS=Homo sapiens GN=CKB PE=1 SV=1 |
| Q9UQ80\|PA2G4_HUMAN | 49.62 | 4 | 1 | 1 | 43787 | Proliferation-associated protein 2G4 OS=Homo sapiens GN=PA2G4 PE=1 SV=3 |
| P12004\|PCNA_HUMAN | 49.16 | 5 | 1 | 1 | 28769 | Proliferating cell nuclear antigen OS=Homo sapiens GN=PCNA PE=1 SV=1 |
| P02545\|LMNA_HUMAN | 49.04 | 1 | 1 | 1 | 74140 | Prelamin-A/C OS=Homo sapiens GN=LMNA PE=1 SV=1 |
| P22681\|CBL_HUMAN | 38.32 | 1 | 1 | 1 | 99633 | E3 ubiquitin-protein ligase CBL OS=Homo sapiens GN=CBL PE=1 SV=2 |
| Q13191\|CBLB_HUMAN | 38.32 | 1 | 1 | 1 | 109450 | E3 ubiquitin-protein ligase CBL-B OS=Homo sapiens GN=CBLB PE=1 SV=2 |
| P62826\|RAN_HUMAN | 38.16 | 5 | 1 | 1 | 24423 | GTP-binding nuclear protein Ran OS=Homo sapiens GN=RAN PE=1 SV=3 |
| P49773\|HINT1_HUMAN | 34.91 | 6 | 1 | 1 | 13802 | Histidine triad nucleotide-binding protein 1 OS=Homo sapiens GN=HINT1 PE=1 SV=2 |
| P02768\|ALBU_HUMAN | 31.64 | 2 | 1 | 1 | 69367 | Serum albumin OS=Homo sapiens GN=ALB PE=1 SV=2 |
| Total 57 proteins |  |  |  |  |  |  |

**Additional file 1: Table S2**. Mass spectrometry analysis of calibrator from SIRPA ELISA kit (Cusabio)

| **Accession** | **-10lgP** | **Coverage (%)** | **#Peptides** | **#Unique** | **Avg. Mass** | **Description** |
| --- | --- | --- | --- | --- | --- | --- |
| P02768\|ALBU_HUMAN | 246.8 | 16 | 19 | 19 | 69367 | Serum albumin OS=Homo sapiens GN=ALB PE=1 SV=2 |
| P02769\|ALBU_BOVIN | 649.89 | 94 | 445 | 445 | 69294 | Serum albumin OS=Bos taurus GN=ALB PE=1 SV=4 |
| Q2UVX4\|CO3_BOVIN | 330.73 | 5 | 34 | 34 | 187252 | Complement C3 OS=Bos taurus GN=C3 PE=1 SV=2 |
| P41361\|ANT3_BOVIN | 222.87 | 6 | 8 | 8 | 52347 | Antithrombin-III OS=Bos taurus GN=SERPINC1 PE=1 SV=2 |
| P01966\|HBA_BOVIN | 208.19 | 29 | 15 | 15 | 15184 | Hemoglobin subunit alpha OS=Bos taurus GN=HBA PE=1 SV=2 |
| Q29443\|TRFE_BOVIN | 149.57 | 6 | 3 | 3 | 77753 | Serotransferrin OS=Bos taurus GN=TF PE=2 SV=1 |
| P01030\|CO4_BOVIN | 97.42 | 3 | 3 | 3 | 101908 | Complement C4 (Fragments) OS=Bos taurus GN=C4 PE=1 SV=2 |
| O46375\|TTHY_BOVIN | 74.69 | 20 | 1 | 1 | 15727 | Transthyretin OS=Bos taurus GN=TTR PE=1 SV=1 |
| P12763\|FETUA_BOVIN | 58.6 | 3 | 1 | 1 | 38419 | Alpha-2-HS-glycoprotein OS=Bos taurus GN=AHSG PE=1 SV=2 |
| P17690\|APOH_BOVIN | 52.02 | 3 | 1 | 1 | 38252 | Beta-2-glycoprotein 1 OS=Bos taurus GN=APOH PE=1 SV=4 |
| P81187\|CFAB_BOVIN | 45.32 | 2 | 2 | 2 | 85366 | Complement factor B OS=Bos taurus GN=CFB PE=1 SV=2 |
| Q1RMK9\|F174B_BOVIN | 44.73 | 4 | 1 | 1 | 16654 | Membrane protein FAM174B OS=Bos taurus GN=FAM174B PE=2 SV=1 |
| Q2YDD9\|ADT4_BOVIN | 43.95 | 2 | 1 | 1 | 35694 | ADP/ATP translocase 4 OS=Bos taurus GN=SLC25A31 PE=2 SV=1 |
| A2I7N0\|SPA34_BOVIN | 37.23 | 2 | 1 | 1 | 46311 | Serpin A3-4 OS=Bos taurus GN=SERPINA3-4 PE=3 SV=1 |
| A2I7N1\|SPA35_BOVIN | 37.23 | 2 | 1 | 1 | 46397 | Serpin A3-5 OS=Bos taurus GN=SERPINA3-5 PE=3 SV=1 |
| Q9TTE1\|SPA31_BOVIN | 37.23 | 2 | 1 | 1 | 46237 | Serpin A3-1 OS=Bos taurus GN=SERPINA3-1 PE=1 SV=3 |
| A2I7M9\|SPA32_BOVIN | 37.23 | 2 | 1 | 1 | 46237 | Serpin A3-2 OS=Bos taurus GN=SERPINA3-2 PE=3 SV=1 |
| Q3ZEJ6\|SPA33_BOVIN | 37.23 | 2 | 1 | 1 | 46326 | Serpin A3-3 OS=Bos taurus GN=SERPINA3-3 PE=1 SV=2 |
| Q2TBI1\|NSE4A_BOVIN | 32.36 | 3 | 1 | 1 | 43728 | Non-structural maintenance of chromosomes element 4 homolog A OS=Bos taurus GN=NSMCE4A PE=2 SV=1 |
| A4FUZ0\|KRT83_BOVIN | 30.29 | 2 | 1 | 1 | 53989 | Keratin, type II cuticular Hb3 OS=Bos taurus GN=KRT83 PE=2 SV=1 |
| Q148H4\|KRT81_BOVIN | 30.29 | 2 | 1 | 1 | 54613 | Keratin, type II cuticular Hb1 OS=Bos taurus GN=KRT81 PE=2 SV=1 |
| Q58CX6\|ATAT_BOVIN | 30.07 | 5 | 1 | 1 | 25675 | Alpha-tubulin N-acetyltransferase OS=Bos taurus GN=ATAT1 PE=2 SV=1 |
| Q58DQ5\|RT09_BOVIN | 29.61 | 2 | 1 | 1 | 45231 | 28S ribosomal protein S9, mitochondrial OS=Bos taurus GN=MRPS9 PE=1 SV=3 |
| E1BP74\|MARF1_BOVIN | 28.37 | 0 | 1 | 1 | 192125 | Meiosis arrest female protein 1 OS=Bos taurus GN=MARF1 PE=3 SV=2 |
| Q3T087\|RL11_BOVIN | 27.71 | 4 | 1 | 1 | 20252 | 60S ribosomal protein L11 OS=Bos taurus GN=RPL11 PE=2 SV=3 |
| Total 25 proteins |  |  |  |  |  |  |

**Additional file 1: Table S3**. Mass spectrometry analysis of calibrator from Elabscience SIRPA ELISA kit

| **Accession** | **-10lgP** | **Coverage (%)** | **#Peptides** | **#Unique** | **Avg. Mass** | **Description** |
| --- | --- | --- | --- | --- | --- | --- |
| P02768\|ALBU_HUMAN | 222.06 | 22 | 35 | 35 | 69367 | Serum albumin OS=Homo sapiens GN=ALB PE=1 SV=2 |
| P02452\|CO1A1_HUMAN | 211.96 | 13 | 23 | 22 | 138942 | Collagen alpha-1(I) chain OS=Homo sapiens GN=COL1A1 PE=1 SV=5 |
| P08123\|CO1A2_HUMAN | 125.57 | 5 | 10 | 10 | 129314 | Collagen alpha-2(I) chain OS=Homo sapiens GN=COL1A2 PE=1 SV=7 |
| P02461\|CO3A1_HUMAN | 94.48 | 2 | 4 | 3 | 138564 | Collagen alpha-1(III) chain OS=Homo sapiens GN=COL3A1 PE=1 SV=4 |
| P04264\|K2C1_HUMAN | 44.59 | 2 | 1 | 1 | 66039 | Keratin, type II cytoskeletal 1 OS=Homo sapiens GN=KRT1 PE=1 SV=6 |
| P02788-2\|TRFL_HUMAN | 40.86 | 1 | 1 | 1 | 73161 | Isoform DeltaLf of Lactotransferrin OS=Homo sapiens GN=LTF |
| P02787\|TRFE_HUMAN | 40.86 | 1 | 1 | 1 | 77064 | Serotransferrin OS=Homo sapiens GN=TF PE=1 SV=3 |
| P02788\|TRFL_HUMAN | 40.86 | 1 | 1 | 1 | 78182 | Lactotransferrin OS=Homo sapiens GN=LTF PE=1 SV=6 |
| P05997\|CO5A2_HUMAN | 33.75 | 1 | 1 | 1 | 144910 | Collagen alpha-2(V) chain OS=Homo sapiens GN=COL5A2 PE=1 SV=3 |
| Q03181-4\|PPARD_HUMAN | 33.65 | 2 | 1 | 1 | 38855 | Isoform 4 of Peroxisome proliferator-activated receptor delta OS=Homo sapiens GN=PPARD |
| Q03181-2\|PPARD_HUMAN | 33.65 | 2 | 1 | 1 | 40406 | Isoform 2 of Peroxisome proliferator-activated receptor delta OS=Homo sapiens GN=PPARD |
| Q03181-3\|PPARD_HUMAN | 33.65 | 2 | 1 | 1 | 45764 | Isoform 3 of Peroxisome proliferator-activated receptor delta OS=Homo sapiens GN=PPARD |
| Q03181\|PPARD_HUMAN | 33.65 | 2 | 1 | 1 | 49903 | Peroxisome proliferator-activated receptor delta OS=Homo sapiens GN=PPARD PE=1 SV=1 |
| Q07869\|PPARA_HUMAN | 33.65 | 1 | 1 | 1 | 52225 | Peroxisome proliferator-activated receptor alpha OS=Homo sapiens GN=PPARA PE=1 SV=2 |
| P07477\|TRY1_HUMAN | 29.55 | 3 | 1 | 1 | 26558 | Trypsin-1 OS=Homo sapiens GN=PRSS1 PE=1 SV=1 |
| Q9BYE2-3\|TMPSD_HUMAN | 29.55 | 2 | 1 | 1 | 57631 | Isoform 3 of Transmembrane protease serine 13 OS=Homo sapiens GN=TMPRSS13 |
| Q9BYE2-5\|TMPSD_HUMAN | 29.55 | 1 | 1 | 1 | 59289 | Isoform 5 of Transmembrane protease serine 13 OS=Homo sapiens GN=TMPRSS13 |
| Q9BYE2-2\|TMPSD_HUMAN | 29.55 | 1 | 1 | 1 | 60807 | Isoform 2 of Transmembrane protease serine 13 OS=Homo sapiens GN=TMPRSS13 |
| Q9BYE2\|TMPSD_HUMAN | 29.55 | 1 | 1 | 1 | 63153 | Transmembrane protease serine 13 OS=Homo sapiens GN=TMPRSS13 PE=2 SV=4 |
| P12107-4\|COBA1_HUMAN | 28.64 | 1 | 1 | 1 | 167752 | Isoform 4 of Collagen alpha-1(XI) chain OS=Homo sapiens GN=COL11A1 |
| P12107-3\|COBA1_HUMAN | 28.64 | 1 | 1 | 1 | 176619 | Isoform C of Collagen alpha-1(XI) chain OS=Homo sapiens GN=COL11A1 |
| P12107\|COBA1_HUMAN | 28.64 | 1 | 1 | 1 | 181064 | Collagen alpha-1(XI) chain OS=Homo sapiens GN=COL11A1 PE=1 SV=4 |
| P12107-2\|COBA1_HUMAN | 28.64 | 1 | 1 | 1 | 182421 | Isoform B of Collagen alpha-1(XI) chain OS=Homo sapiens GN=COL11A1 |
| Q5CZC0\|FSIP2_HUMAN | 22.12 | 0 | 1 | 1 | 780624 | Fibrous sheath-interacting protein 2 OS=Homo sapiens GN=FSIP2 PE=2 SV=4 |
| Q8WWM7-6\|ATX2L_HUMAN | 22.07 | 1 | 1 | 1 | 102895 | Isoform 6 of Ataxin-2-like protein OS=Homo sapiens GN=ATXN2L |
| Q8WWM7-8\|ATX2L_HUMAN | 22.07 | 1 | 1 | 1 | 110143 | Isoform 8 of Ataxin-2-like protein OS=Homo sapiens GN=ATXN2L |
| Q8WWM7-4\|ATX2L_HUMAN | 22.07 | 1 | 1 | 1 | 110326 | Isoform 4 of Ataxin-2-like protein OS=Homo sapiens GN=ATXN2L |
| Q8WWM7-5\|ATX2L_HUMAN | 22.07 | 1 | 1 | 1 | 110177 | Isoform 5 of Ataxin-2-like protein OS=Homo sapiens GN=ATXN2L |
| Q8WWM7-9\|ATX2L_HUMAN | 22.07 | 1 | 1 | 1 | 112091 | Isoform 9 of Ataxin-2-like protein OS=Homo sapiens GN=ATXN2L |
| Q8WWM7-2\|ATX2L_HUMAN | 22.07 | 1 | 1 | 1 | 111920 | Isoform 2 of Ataxin-2-like protein OS=Homo sapiens GN=ATXN2L |
| Q8WWM7\|ATX2L_HUMAN | 22.07 | 1 | 1 | 1 | 113374 | Ataxin-2-like protein OS=Homo sapiens GN=ATXN2L PE=1 SV=2 |
| Q8WWM7-3\|ATX2L_HUMAN | 22.07 | 1 | 1 | 1 | 115582 | Isoform 3 of Ataxin-2-like protein OS=Homo sapiens GN=ATXN2L |
| Q2M389-2\|WASH7_HUMAN | 20.43 | 1 | 1 | 1 | 98765 | Isoform 2 of WASH complex subunit 7 OS=Homo sapiens GN=KIAA1033 |
| Q2M389\|WASH7_HUMAN | 20.43 | 1 | 1 | 1 | 136403 | WASH complex subunit 7 OS=Homo sapiens GN=KIAA1033 PE=1 SV=2 |
| sp\|P02769\|ALBU_BOVIN | 473.99 | 95 | 325 | 322 | 69294 | Serum albumin OS=Bos taurus GN=ALB PE=1 SV=4 |
| sp\|P02453\|CO1A1_BOVIN | 199.58 | 12 | 26 | 25 | 138939 | Collagen alpha-1(I) chain OS=Bos taurus GN=COL1A1 PE=1 SV=3 |
| sp\|P00760\|TRY1_BOVIN | 183.41 | 37 | 11 | 11 | 25785 | Cationic trypsin OS=Bos taurus PE=1 SV=3 |
| sp\|P02465\|CO1A2_BOVIN | 157.14 | 7 | 13 | 13 | 129064 | Collagen alpha-2(I) chain OS=Bos taurus GN=COL1A2 PE=1 SV=2 |
| sp\|Q3SZR3\|A1AG_BOVIN | 135.06 | 31 | 6 | 6 | 23182 | Alpha-1-acid glycoprotein OS=Bos taurus GN=ORM1 PE=2 SV=1 |
| tr\|Q5GN72\|Q5GN72_BOVIN | 135.06 | 31 | 6 | 6 | 23158 | Alpha-1 acid glycoprotein (Precursor) OS=Bos taurus GN=agp PE=2 SV=2 |
| tr\|F1N514\|F1N514_BOVIN | 107.69 | 12 | 5 | 5 | 50338 | Uncharacterized protein OS=Bos taurus GN=CD5L PE=4 SV=2 |
| tr\|F1N2Y4\|F1N2Y4_BOVIN | 91.28 | 4 | 4 | 3 | 93843 | Collagen alpha-1(III) chain (Fragment) OS=Bos taurus GN=COL3A1 PE=4 SV=2 |
| tr\|F1MXS8\|F1MXS8_BOVIN | 91.28 | 3 | 4 | 3 | 138928 | Collagen alpha-1(III) chain OS=Bos taurus GN=COL3A1 PE=4 SV=2 |
| sp\|P04258\|CO3A1_BOVIN | 91.28 | 4 | 4 | 3 | 93651 | Collagen alpha-1(III) chain OS=Bos taurus GN=COL3A1 PE=1 SV=1 |
| tr\|F1MSZ6\|F1MSZ6_BOVIN | 82.5 | 5 | 2 | 2 | 52440 | Antithrombin-III OS=Bos taurus GN=SERPINC1 PE=3 SV=1 |
| sp\|P41361\|ANT3_BOVIN | 82.5 | 5 | 2 | 2 | 52347 | Antithrombin-III OS=Bos taurus GN=SERPINC1 PE=1 SV=2 |
| sp\|P81644\|APOA2_BOVIN | 77.76 | 17 | 2 | 2 | 11202 | Apolipoprotein A-II OS=Bos taurus GN=APOA2 PE=1 SV=2 |
| sp\|O46375\|TTHY_BOVIN | 76.47 | 20 | 2 | 2 | 15727 | Transthyretin OS=Bos taurus GN=TTR PE=1 SV=1 |
| sp\|Q29443\|TRFE_BOVIN | 75.31 | 4 | 3 | 3 | 77753 | Serotransferrin OS=Bos taurus GN=TF PE=2 SV=1 |
| tr\|G3X6N3\|G3X6N3_BOVIN | 75.31 | 4 | 3 | 3 | 77666 | Serotransferrin OS=Bos taurus GN=TF PE=4 SV=1 |
| tr\|G3MYZ3\|G3MYZ3_BOVIN | 72.31 | 7 | 4 | 1 | 69562 | Uncharacterized protein OS=Bos taurus GN=AFM PE=4 SV=1 |
| tr\|A5PJE3\|A5PJE3_BOVIN | 66.96 | 2 | 2 | 2 | 66998 | Fibrinogen alpha chain OS=Bos taurus GN=FGA PE=2 SV=1 |
| sp\|P02672\|FIBA_BOVIN | 66.96 | 2 | 2 | 2 | 67012 | Fibrinogen alpha chain OS=Bos taurus GN=FGA PE=1 SV=5 |
| sp\|Q7SIH1\|A2MG_BOVIN | 50.13 | 1 | 1 | 1 | 167575 | Alpha-2-macroglobulin OS=Bos taurus GN=A2M PE=1 SV=2 |
| sp\|P00735\|THRB_BOVIN | 48.88 | 2 | 1 | 1 | 70506 | Prothrombin OS=Bos taurus GN=F2 PE=1 SV=2 |
| sp\|P34955\|A1AT_BOVIN | 45.57 | 4 | 1 | 1 | 46104 | Alpha-1-antiproteinase OS=Bos taurus GN=SERPINA1 PE=1 SV=1 |
| tr\|G5E604\|G5E604_BOVIN | 43.86 | 14 | 1 | 1 | 11058 | Uncharacterized protein (Fragment) OS=Bos taurus PE=4 SV=1 |
| tr\|G3N2D7\|G3N2D7_BOVIN | 43.86 | 13 | 1 | 1 | 12112 | Uncharacterized protein (Fragment) OS=Bos taurus GN=IGLL1 PE=4 SV=1 |
| tr\|G5E5H2\|G5E5H2_BOVIN | 43.86 | 11 | 1 | 1 | 14451 | Uncharacterized protein OS=Bos taurus PE=4 SV=1 |
| tr\|G5E5V1\|G5E5V1_BOVIN | 43.86 | 11 | 1 | 1 | 14478 | Uncharacterized protein OS=Bos taurus PE=4 SV=1 |
| tr\|F1MLW7\|F1MLW7_BOVIN | 43.86 | 6 | 1 | 1 | 24397 | Uncharacterized protein OS=Bos taurus GN=IGLL1 PE=4 SV=2 |
| tr\|F1N160\|F1N160_BOVIN | 43.39 | 7 | 1 | 1 | 26730 | Uncharacterized protein OS=Bos taurus PE=4 SV=2 |
| tr\|A5D7M6\|A5D7M6_BOVIN | 43.35 | 2 | 1 | 1 | 62683 | KRT5 protein OS=Bos taurus GN=KRT5 PE=2 SV=1 |
| sp\|Q5XQN5\|K2C5_BOVIN | 43.35 | 2 | 1 | 1 | 62937 | Keratin, type II cytoskeletal 5 OS=Bos taurus GN=KRT5 PE=1 SV=1 |
| sp\|Q29S21\|K2C7_BOVIN | 43.35 | 3 | 1 | 1 | 51578 | Keratin, type II cytoskeletal 7 OS=Bos taurus GN=KRT7 PE=2 SV=1 |
| sp\|Q08D91\|K2C75_BOVIN | 43.35 | 2 | 1 | 1 | 59036 | Keratin, type II cytoskeletal 75 OS=Bos taurus GN=KRT75 PE=2 SV=1 |
| tr\|G3MXL3\|G3MXL3_BOVIN | 43.35 | 2 | 1 | 1 | 62892 | Uncharacterized protein (Fragment) OS=Bos taurus GN=KRT3 PE=3 SV=1 |
| sp\|P81187\|CFAB_BOVIN | 33.34 | 1 | 1 | 1 | 85366 | Complement factor B OS=Bos taurus GN=CFB PE=1 SV=2 |
| tr\|F1N2Y2\|F1N2Y2_BOVIN | 31.8 | 1 | 1 | 1 | 145159 | Uncharacterized protein OS=Bos taurus GN=COL5A2 PE=4 SV=2 |
| tr\|G3MXE4\|G3MXE4_BOVIN | 29.53 | 1 | 1 | 1 | 123141 | Uncharacterized protein (Fragment) OS=Bos taurus GN=COL22A1 PE=4 SV=1 |
| tr\|E1BG60\|E1BG60_BOVIN | 29.53 | 1 | 1 | 1 | 161189 | Uncharacterized protein OS=Bos taurus GN=COL22A1 PE=4 SV=2 |
| tr\|F1N0K0\|F1N0K0_BOVIN | 27.34 | 1 | 1 | 1 | 182353 | Collagen alpha-1(XI) chain OS=Bos taurus GN=COL11A1 PE=4 SV=2 |
| tr\|G5E5D5\|G5E5D5_BOVIN | 22.37 | 0 | 1 | 1 | 771059 | Uncharacterized protein OS=Bos taurus PE=4 SV=1 |
| tr\|F1MB15\|F1MB15_BOVIN | 22.37 | 0 | 1 | 1 | 770968 | Uncharacterized protein OS=Bos taurus GN=FSIP2 PE=4 SV=2 |
| tr\|E1BH49\|E1BH49_BOVIN | 22.37 | 0 | 1 | 1 | 771319 | Uncharacterized protein OS=Bos taurus GN=FSIP2 PE=4 SV=2 |
| tr\|G5E600\|G5E600_BOVIN | 20.9 | 1 | 1 | 1 | 136503 | Uncharacterized protein OS=Bos taurus GN=KIAA1033 PE=4 SV=1 |
| tr\|F1MW05\|F1MW05_BOVIN | 20.59 | 1 | 1 | 1 | 84092 | Uncharacterized protein (Fragment) OS=Bos taurus GN=LOC510388 PE=3 SV=2 |
| tr\|F1MLB1\|F1MLB1_BOVIN | 20.23 | 0 | 1 | 1 | 341154 | Uncharacterized protein OS=Bos taurus PE=4 SV=2 |
| Total 78 proteins |  |  |  |  |  |  |
